# Obesity and adverse childhood experiences in relation to stress during the COVID-19 pandemic: an analysis of the Canadian Longitudinal Study on Aging

**DOI:** 10.1038/s41366-023-01258-9

**Published:** 2023-01-23

**Authors:** Vanessa De Rubeis, Andrea Gonzalez, Margaret de Groh, Ying Jiang, Urun Erbas Oz, Jean-Eric Tarride, Nicole E. Basta, Susan Kirkland, Christina Wolfson, Lauren E. Griffith, Parminder Raina, Laura N. Anderson, the Canadian Longitudinal Study on Aging (CLSA) Team, Andrew Costa, Cynthia Balion, Yukiko Asada, Benoȋt Cossette, Melanie Levasseur, Scott Hofer, Theone Paterson, David Hogan, Jacqueline McMillan, Teresa Liu-Ambrose, Verena Menec, Philip St. John, Gerald Mugford, Zhiwei Gao, Vanessa Taler, Patrick Davidson, Andrew Wister, Theodore Cosco

**Affiliations:** 1 Department of Health Research Methods, Evidence, and Impact, McMaster University, Hamilton, ON L8S 4L8 Canada; 2 Department of Psychiatry & Behavioural Neurosciences, Offord Centre for Child Studies, McMaster University, Hamilton, ON L8S 4K1 Canada; 3 Applied Research Division, Center for Surveillance and Applied Research, Public Health Agency of Canada, Ottawa, ON K0A 0K9 Canada; 4 McMaster University, Chair in Health Technology Management, Hamilton, ON L8S 4L8 Canada; 5 Center for Health Economics and Policy Analysis (CHEPA), McMaster University, Hamilton, ON L8S 4L8 Canada; 6 Programs for Assessment of Technology in Health (PATH), The Research Institute of St. Joe’s Hamilton, St. Joseph’s Healthcare Hamilton, Hamilton, ON L8N 4A6 Canada; 7 Department of Epidemiology, Biostatistics and Occupational Health, School of Population and Global Health, McGill University, Montreal, QC H3A 1A2 Canada; 8 Department of Community Health & Epidemiology and Division of Geriatric Medicine, Dalhousie University, Halifax, NS B3H 4R2 Canada; 9 Department of Medicine, Faculty of Health Sciences, McGill University, Montreal, QC H3A 1A2 Canada; 10 Research Institute of the McGill University Health Centre, McGill University, Montreal, QC H3A 1A2 Canada; 11 Faculty of Medicine and Health Sciences, University of Sherbrooke, Sherbrooke, QC Canada; 12 Department of Psychology, University of Victoria, Victoria, BC Canada; 13 Faculty of Medicine, University of Calgary, Calgary, AB Canada; 14 Faculty of Medicine, University of British Columbia, Vancouver, BC Canada; 15 Max Rady College of Medicine, University of Manitoba, Winnipeg, MB Canada; 16 Faculty of Medicine, Memorial University of Newfoundland, St. John’s, NL Canada; 17 School of Psychology, University of Ottawa, Ottawa, ON Canada; 18 Department of Gerontology, Simon Fraser University, Vancouver, BC Canada

**Keywords:** Risk factors, Epidemiology

## Abstract

**Background:**

People with obesity are at increased risk of chronic stress, and this may have been exacerbated during the COVID-19 pandemic. Adverse childhood experiences (ACE) are also associated with both obesity and stress, and may modify risk of stress among people with obesity. The objectives of this study were to evaluate the associations between obesity, ACEs, and stress during the pandemic, and to determine if the association between obesity and stress was modified by ACEs.

**Methods:**

A longitudinal study was conducted among adults aged 50–96 years (*n* = 23,972) from the Canadian Longitudinal Study on Aging (CLSA) COVID-19 Study. Obesity and ACEs were collected pre-pandemic (2015–2018), and stress was measured at COVID-19 Exit Survey (Sept-Dec 2020). We used logistic, Poisson, and negative binomial regression to estimate relative risks (RRs) and 95% confidence intervals (CIs) for the associations between obesity, ACEs, and stress outcomes during the pandemic. Interaction by ACEs was evaluated on the additive and multiplicative scales.

**Results:**

People with obesity were more likely to experience an increase in overall stressors (class III obesity vs. healthy weight RR = 1.19; 95% CI: 1.12–1.27) as well as increased health related stressors (class III obesity vs. healthy weight RR: 1.25; 95% CI: 1.12–1.39) but did not perceive the consequences of the pandemic as negative. ACEs were also associated an increase in overall stressors (4–8 ACEs vs. none RR = 1.38; 95% CI: 1.33–1.44) and being more likely to perceive the pandemic as negative (4–8 ACEs vs. none RR = 1.32; 95% CI: 1.19–1.47). The association between obesity and stress was not modified by ACEs.

**Conclusions:**

Increased stress during the first year of the COVID-19 pandemic was observed among people with obesity or ACEs. The long-term outcomes of stress during the pandemic need to be determined.

## Introduction

Stress and obesity share a complex relationship, with cyclical and bidirectional associations across the life course [1, 2]. As described in a conceptual model by van der Valk et al., the bidirectional interplay between obesity, chronic stress, and glucocorticoid action is impacted by numerous individual level characteristics, including genetics, lifestyle, medications and mental distress [1]. “It is well known that stress and obesity are associated”, and many mechanistic pathways that lead to disease development exist, including health behaviors, glucocorticoid activation, and mental health [1, 3, 4]. However, having obesity has also been found to increase stress due to several reasons, including comorbidities that limit daily activities, and weight stigma or bias, which may induce a prolonged stress response [3]. Adverse childhood experiences (ACEs), defined as a wide range of negative events, including abuse, neglect, witnessing violence, parental mental illness or incarceration of a family member [5], are one example of an individual level factor that may impact both obesity and stress [4–8]. Although ACEs take place early in life, the effects have been found to extend beyond childhood or adolescence into older adulthood [4]. Following exposure to ACEs there may be a prolonged stress response, which is also known as toxic stress, which may make people with ACEs more susceptible to stress or worse experiences during a stressful event [5–7, 9].

In Canada, throughout the first year of the pandemic (March 2020-March 2021), strict public health preventive measures were in place including work and school closures, and travel bans [10]. In turn, this created wide-reaching implications on population health including an increase in stress [11–13]. Obesity was identified as a risk factor for increased COVID-19 morbidity and mortality early in the pandemic and this may have contributed to increased weight bias and stress among people living with obesity [14–16]. There is limited research on the impact of living with obesity on stress during the COVID-19 pandemic. A systematic review has found that exposure to disasters increases cardiometabolic risk, including obesity, across the life course, however, research has not yet explored how obesity may influence stress experienced during a disaster [17]. Stress during a disaster can be measured objectively, using reports of stressors, or subjectively, measuring perceptions of a disaster [18]. ACEs are an established risk factor for both stress [5–7] and later life obesity [4, 8]. ACEs have been associated with higher psychological symptoms following a natural disaster [19] and it is possible people with obesity who experienced ACEs may have experienced an added burden of stress during the pandemic [20–22]. Differences also exist by sex, whereby females have higher psychological symptoms following a disaster [19], and the prevalence of ACEs and obesity vary among males and females [23, 24].

It is possible people with obesity may have experienced greater stress during the COVID-19 pandemic, and life course epidemiology frameworks help to identify potential distal risk factors, such as ACEs, that may have led to differential experiences [25]. This is of importance in the context of the pandemic, and beyond, as the findings from this research will contribute to understanding the complex relationship stress and obesity share. The objectives of this study were to evaluate the association between both obesity and ACEs and stress during the pandemic (stressors and perceived consequences) and to determine if the association between obesity and stress was modified by ACEs. Differences in the association between obesity and ACEs by sex were also evaluated.

## Methods

### Study design and participants

We conducted an analysis using longitudinal data collected as part of the Canadian Longitudinal Study on Aging (CLSA). The methodology of the CLSA has been published elsewhere [26]. Briefly, the CLSA is a national longitudinal study of adults aged 45–85 at the time of recruitment (2011–2015). At the time of recruitment, participants provided informed consent. Adults from the 10 Canadian provinces were recruited using population-based sampling strategies [26]. Participants were eligible for inclusion into the CLSA if they could complete interviews in English or French, were cognitively able participate on their own, were not in an institution, did not reside in a Canadian territory (The Northwest Territories, the Yukon and Nunavut) or on a Federal First Nations reserve, and were not a full-time member of the Canadian Armed forces. Ethics approval for this study was received from the Hamilton Integrated Research Ethics Board (HiREB).

The CLSA is comprised of the Tracking cohort and the Comprehensive cohort. Data for the Tracking cohort were collected using telephone interviews, whereas data for the Comprehensive cohort were collected via in-home interviews and clinical data collection site visits. Data are collected every three years and all participants will be followed for 20 years, or until death or loss-to-follow-up. Data for this analysis were collected at baseline in 2011–2015 and at follow-up 1 in 2015–2018. At the start of the COVID-19 pandemic, the CLSA COVID-19 Questionnaire Study was implemented, which collected longitudinal data from April 2020 to December 2020. Specific details about when data for this study were collected can be found in Table 1.

**Table 1:** Characteristics of participants from the Canadian Longitudinal Study on Aging (CLSA) COVID-19 Questionnaire study (*n* = 23,972), Canada.

Characteristics	*N* (%) (*n* = 23,972)
Sex^a^	
Male	11,229 (47%)
Female	12,743 (53%)
Age group^b^	
50–64 years	8347 (35%)
65–74 years	8759 (36%)
75–96 years	6866 (29%)
Racial background^a^	
White	23,273 (97%)
Non-white	673 (3%)
Missing	26
Total household income^c^	
Less than $50,000	5716 (25%)
$50,000 to less than $100,000	8569 (38%)
$100,000 to less than $150,000	4589 (20%)
$150,000 or more	3758 (17%)
Missing	1340
CESD-10 score ≥10^c^	
No	20,548 (87%)
Yes	3096 (13%)
Missing	328
Alcohol consumption^c^	
Did not drink in last 12 months	2777 (12%)
Occasional drinker	2856 (12%)
Regular drinker (at least once a month)	18,312 (76%)
Missing	27
Physical activity^c^	
≤150 min/week of moderate-intensity or ≤ 75 min/week of vigorous intensity activity (high risk)	16,473 (69%)
>150 min/week of moderate-intensity or >75 min/week of vigorous-intensity activity (low risk)	7485 (31%)
Missing	14
Number of ACEs	
0	9253 (39%)
1	6566 (28%)
2	3652 (15%)
3	2152 (9%)
4–8	2237 (9%)
Missing	112
Body mass index^c^	
Normal weight (≤24.9 kg/m^2^)	6710 (28%)
Overweight (25.0–29.9 kg/m^2^)	9748 (41%)
Obesity—Class I (30.0–34.9 kg/m^2^)	4779 (20%)
Obesity—Class II (35.0–39.9 kg/m^2^)	1674 (7%)
Obesity—Class III ( ≥ 40.0 kg/m^2^)	835 (4%)
Missing	226

*ACEs* adverse childhood experiences, *CESD-10* center for epidemiologic studies short depression scale, *kg* kilogram, *m* meters.

^a^Data collected at CLSA Baseline (2011–2015).

^b^Data collected at CLSA COVID-19 Questionnaire Baseline Survey (April 2020-June 2020).

^c^Data collected at CLSA Follow-up 1 (2015–2018).

### Primary exposures

#### Obesity

Obesity was measured at CLSA follow-up 1 (2015–2018). For individuals in the Comprehensive cohort (*n* = 15,582), height and weight were measured by trained research assistants. These measurements were used to calculate body mass index (BMI) (kg/m^2^). For individuals in the Tracking cohort (*n* = 8390), height and weight were assessed using self-report, which were then used to calculate BMI. A correction factor developed by Statistics Canada was applied to the self-reported BMI to account for bias associated with self-report [27]. These correction equations were generated using the 2005 Canadian Community Health Survey with consideration of several sociodemographic variables separately for males and females [28]. Self-reported BMI was slightly underestimated compared to the corrected BMI, which is consistent with the literature [27]. BMI was categorized following World Health Organization standard cut-offs [29]: normal weight (≤24.9 kg/m^2^), overweight (25–29.9 kg/m^2^), obesity class I (30–34.9 kg/m^2^), obesity class II (35–39.9 kg/m^2^) and obesity class III (≥40 kg/m^2^). Obesity was further classified into 3 subgroups, as research has found variation in risk of health outcomes across the subtypes [30]. Underweight individuals were included in the normal weight category given the small sample size.

#### Adverse childhood experiences

To measure ACEs, at CLSA follow-up 1, participants were asked about 11 experiences before the age of 16 related to physical abuse, sexual abuse, emotional abuse, neglect, and exposure to intimate partner violence. Participants were also asked about three experiences before the age of 18 related to death of a parent, parental divorce/separation and living with a family member with mental health problems. These questions were adapted from the Childhood Experience of Violence Questionnaire and the National Longitudinal Study of Adolescent to Adult Health Wave III questionnaire [31, 32]. Based on responses to dichotomized yes/no questions, a cumulative score was created by summing the total number of ACEs participants reported [31]. Since only a small proportion of people reported 5 to 8 ACEs (4%), those reporting four or more were collapsed into one group. A cumulative ACEs score was used rather than subgroups by severity, as research has found this to be a better assessment of cumulative exposure, and has been found to be associated with health outcomes [33].

### Measurement of outcomes (stress)

Stress was measured in two ways: (1) stressors and (2) the perceived consequences of the pandemic. These questions have previously been used in disaster research [18, 34–36] to study objective and subjective stress following a disaster such as the COVID-19 pandemic. The development of these questions were modified from gold-standard measurement tools [18].

#### Stressors

Stressors were measured at CLSA COVID-19 Questionnaire Study Exit Survey (September 2020-December 2020). Participants were asked, “*Which of the following have you experienced during the COVID-19 pandemic?”* where participants could select one or more of the following options: participant was ill, someone close to the participant was ill, someone close to the participant died, loss of income, unable to access necessary food and supplies, unable to access healthcare, unable to access usual prescriptions, increased conflict, separation from family, increased caregiving, unable to care for those who require assistance due to limitations, and breakdown in family relationships. The 12 stressors were classified into four domains for this analysis; (1) health (participant was ill, someone close to the participant was ill, someone close to the participant died), (2) resources (loss of income, unable to access necessary food and supplies, unable to access healthcare, unable to access usual prescriptions), (3) relationships (increased conflict, separation from family, breakdown in family relationships), and (4) caregiving (increased caregiving, unable to care for those who require assistance due to limitations). To create each domain, the total number of stressors within each category was summed. The range of values for each domain varied depending on how many stressors fell within the category. For instance, the health domain ranged from 0 to 3, whereas the resources domain ranged from 0 to 4. In addition, a cumulative stressor score was created by summing the total number of stressors participants experienced across all domains [37]. The cumulative stressor score ranged from 0 to 12.

#### Perceived consequences of the pandemic

As a subjective measure of perceived stress, participants were asked “Taking everything about COVID-19 into account, how would you describe the consequences of COVID-19 on you and your household?” during the CLSA COVID-19 Questionnaire Study Exit Survey (September 2020-December 2020) [18, 34, 35]. Response options were very negative, negative, neutral, positive, and very positive. Very few participants reported the consequences of the pandemic as very negative or very positive, so these categories were combined with negative and positive response options, respectively. The neutral category was further combined with the positive and very positive category to create a binary variable, since the objective of the analysis was to explore negative/very negative perceived consequences of the pandemic compared to all other perceptions.

### Measurement of potential confounding variables

All remaining variables were measured at CLSA baseline (2011–2015), CLSA follow-up 1 (2015–2018), CLSA COVID-19 Baseline Survey (April 2020-June 2020) or the CLSA COVID-19 Exit Survey (September 2020-December 2020). These variables were chosen based on the framework proposed by van der Valk et al., identifying characteristics that are related to the association of stress and obesity [1]. Participant sex (male or female) and racial background (white or non-white) were collected at CLSA baseline. Participant age at CLSA COVID-19 Baseline Survey was categorized as 50–64 years, 65–74 years, and 75–96 years. Physical activity, household income, alcohol consumption and depression were measured at CLSA follow-up 1. The Physical Activity Scale for the Elderly (PASE) was used to assess level of physical activity for the previous seven days [38]. Based on the World Health Organization (WHO) guidelines [39], physical activity was dichotomized into ≤150 min/week of moderate-intensity or ≤75 min/week of vigorous-intensity versus >150 min/week of moderate-intensity or >75 min/week of vigorous-intensity. Household income was categorized into less than $50,000, $50,000 to less than $100,000, $100,000 to less than $150,000, and $150,000 or more, and alcohol consumption over the past 12 months was categorized as did not drink in the last 12 months, occasional drinker, and regular drinker (at least once a month). Depression was assessed using the Center for Epidemiologic Studies Short Depression (CESD) scale [40], where a score of ≥10 indicates risk for clinical depression.

### Statistical analysis

All statistical analyses were completed using SAS 9.4. Statistical code is available upon request. The associations between both obesity and ACEs were independently evaluated in relation to the three primary outcomes, (1) the stressor domains, (2) total stressor score, and (3) the perceived consequences of the pandemic. PROC GENMOD was used to estimate relative risks (RRs) and 95% confidence intervals (CIs). For all outcomes, a log link function was used, however the distribution used varied for different outcomes. For the stressor domains, a Poisson distribution was assumed as this was a count variable. Although the total stressor variable was also a count variable, a negative binomial distribution was assumed given the overdispersion. Finally, a binomial distribution was assumed for the binary perceived consequences of the pandemic variable. All models were adjusted for potential confounders that were hypothesized a priori to be predictors of both the exposures and outcome variables. These included sex, age group, racial background, physical activity, household income, alcohol consumption and depression [1]. For the association between ACEs and stress, an additional model was run adding obesity to the fully adjusted model, given the potential mediating role of obesity. All variables had less than 5% of participants missing, and a complete case analysis was conducted. A sensitivity analysis was conducted to explore differences in associations by severity of ACEs. We explored the association between maltreatment ACEs and measures of stress, and family dysfunction ACEs and measures of stress.

For the association between obesity and measures of stress, interaction by both ACEs and sex were assessed separately on both the additive and multiplicative scales. In epidemiologic research, interaction is often only explored on the multiplicative scale, however, the assessment of interaction on the additive scale has significant public health importance as it can contribute to better allocation of resources and identification of high-risk subgroups [41]. STROBE guidelines recommend presenting the separate effects of exposures and modifiers, as well as joint effects to ensure readers can assess interaction on either scale [41]. To determine if the associations between obesity and measures of stress were modified by ACEs, a dichotomous ACEs variable was created. Individuals who reported no ACEs were categorized as none, and those who reported one or more ACES, were categorized as yes. Using the framework proposed by Knol and VanderWeele [41], interaction was tested on the additive scale using the relative excess risk due to interaction (RERI) and on the multiplicative scale using the ratio of relative risk (RRR). The 95% CI for the RERI were calculated using the delta method [41–43].

## Results

**Fig. 1:**
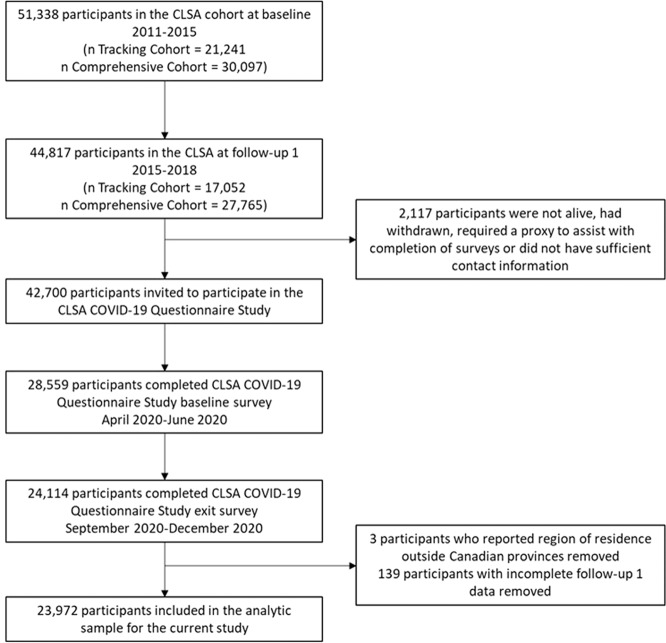
Flowchart of Canadian Longitudinal Study on Aging (CLSA) participants who completed CLSA COVID-19 Questionnaire Exit Survey (September-December 2020).

A total of 23,972 participants were included in this analysis. A detailed flowchart of the analytic sample can be found in Fig. 1 and characteristics of the study population are presented in Table 1. The CLSA COVID-19 Questionnaire Study participants are generally comparable to the full CLSA sample, however, this subgroup had a slightly higher mean household income and higher education than the full sample [44]. Over three quarters (76%) of the participants reported at least one stressor and 63% reported perceiving the consequences of the pandemic as negative or very negative (Table 2).

**Table 2:** Measures of stress during the COVID-19 pandemic among participants from the Canadian Longitudinal Study on Aging (CLSA) COVID-19 Questionnaire study (*n* = 23,972) measured at CLSA COVID-19 Exit survey (Sept 2020–Dec 2020).

	*N* (%) (*n* = 23,972)
Total reported stressors	
0	5781 (24%)
1	6856 (29%)
2	5325 (22%)
3	3135 (13%)
4	1508 (7%)
5	673 (3%)
6	279 (1%)
7–12	201 (1%)
Missing	214
Health domain	
0	16,098 (68%)
1	5630 (24%)
2	1750 (7%)
3	280 (1%)
Missing	214
Resources domain	
0	15,712 (66%)
1	5947 (25%)
2	1656 (7%)
3	383 (2%)
4	60 (0.3%)
Missing	214
Relationship domain	
0	10,916 (46%)
1	11,444 (48%)
2	1170 (5%)
3	228 (1%)
Missing	214
Caregiving domain	
0	19,746 (83%)
1	3595 (15%)
2	417 (2%)
Missing	214
Perceived consequences of the pandemic	
Negative/Very Negative	14,520 (63%)
Neutral/Positive/Very positive	8500 (37%)
Missing	952

### Obesity and stress

A consistent dose-response association was observed for the association between obesity and total stressors, and the health and resources domains, whereby as obesity level increased, the risk of reporting an additional stressor also increased. For instance, adults with class III severe obesity (≥40 kg/m^2^), compared to normal weight (≤24.9 kg/m^2^), had a greater risk of reporting an additional stressor for the total number of stressors (adjusted RR: 1.19; 95% CI: 1.12, 1.27), within the health domain (adjusted RR: 1.25; 95% CI: 1.12, 1.39), and within the resources domain (adjusted RR: 1.38; 95% CI: 1.25, 1.53) (Table 3). Obesity was slightly associated with an increased risk of perceiving the consequences of the pandemic as negative/very negative (obesity class III adjusted RR: 1.05; 95% CI: 1.00, 1.11) (Table 3). When ACEs were added to the model of obesity and stress the results did not change suggesting that ACEs was not a confounder of this association. Results only slighted changed after adjustment for confounding variables (Supplemental Table A1).

**Table 3:** Adjusted relative risks (RRs) and 95% confidence intervals (CIs) for the associations between adverse childhood experiences (ACEs), obesity and measures of stress during the COVID-19 pandemic among older adults in the Canadian Longitudinal Study on Aging (CLSA) COVID-19 Questionnaire Exit Survey.

	Stressor domains				Total number of stressors (*n* = 22,052)	Perceived consequences of the pandemic (*n* = 21,396)
	Health (*n* = 22,052)	Resources (*n* = 22,052)	Relationships (*n* = 22,052)	Caregiving (*n* = 22,052)		
	Adjusted^1^ RR (95% CI)	Adjusted^1^ RR (95% CI)	Adjusted^1^ RR (95% CI)	Adjusted^1^ RR (95% CI)	Adjusted^1^ RR (95% CI)	Adjusted^1^ RR (95% CI)
Number of ACEs						
0	1.00	1.00	1.00	1.00	1.00	1.00
1	1.17 (1.11, 1.23)	1.08 (1.02, 1.13)	1.07 (1.02, 1.12)	1.09 (1.01, 1.18)	1.10 (1.07, 1.13)	1.08 (1.00, 1.15)
2	1.22 (1.15, 1.30)	1.17 (1.10, 1.24)	1.11 (1.05, 1.16)	1.13 (1.03, 1.24)	1.15 (1.11, 1.19)	1.08 (0.99, 1.18)
3	1.38 (1.28, 1.48)	1.30 (1.22, 1.40)	1.15 (1.08, 1.22)	1.22 (1.09, 1.36)	1.25 (1.20, 1.31)	1.22 (1.10, 1.36)
4–8	1.39 (1.30, 1.49)	1.53 (1.43, 1.63)	1.24 (1.17, 1.32)	1.44 (1.20, 1.59)	1.38 (1.33, 1.44)	1.32 (1.19, 1.47)
Obesity						
	Stressor domains				Total number of stressors (*n* = 21,932)	Perceived consequences of the pandemic (*n* = 21,301)
	Health (*n* = 21,932)	Resources (*n* = 21,932)	Relationships (*n* = 21,932)	Caregiving (*n* = 21,932)		
	Adjusted^1^ RR (95% CI)	Adjusted^1^ RR (95% CI)	Adjusted^1^ RR (95% CI)	Adjusted^1^ RR (95% CI)	Adjusted^1^ RR (95% CI)	Adjusted^1^ RR (95% CI)
Normal weight	1.00	1.00	1.00	1.00	1.00	1.00
Overweight	1.03 (0.98, 1.08)	1.05 (1.00, 1.11)	1.01 (0.97, 1.05)	1.01 (0.94, 1.09)	1.02 (0.99, 1.05)	0.97 (0.95, 1.00)
Obesity class I	1.13 (1.07, 1.20)	1.21 (1.14, 1.28)	1.04 (0.99, 1.09)	1.04 (0.95, 1.14)	1.11 (1.07, 1.15)	0.97 (0.94, 1.00)
Obesity class II	1.14 (1.04, 1.23)	1.31 (1.22, 1.42)	1.03 (0.96, 1.10)	1.12 (0.98, 1.26)	1.14 (1.09, 1.20)	1.00 (0.96, 1.04)
Obesity class III	1.25 (1.12, 1.39)	1.38 (1.25, 1.53)	1.04 (0.95, 1.14)	1.08 (0.91, 1.27)	1.19 (1.12, 1.27)	1.05 (1.00, 1.11)

Adjusted for sex, age group, racial background, physical activity, household income, alcohol consumption and depression.

*CI* confidence intervals, *RR* relative risk.

### Adverse childhood experiences and stress

There were 14,607 (61%) adults who experienced one or more ACE (Table 1). Across all outcomes, there was a strong dose-response association, whereby as the number of ACEs increased, the risk of reporting an additional stressor and within each stressor domain also increased (Table 3). For instance, among those with the highest number of ACEs, the risk of an additional stressor within the resources domain was 53% greater than for those with no ACEs (adjusted RR: 1.53; 95% CI: 1.43, 1.63). Similarly, the adjusted RR estimates for the risk of perceiving the consequences of the pandemic as negative/very negative also increased as the number of ACEs increased (Table 3). Those with 4 to 8 ACEs, compared to none, were 32% more likely to perceive the pandemic as negative/very negative compared to neutral/positive/very positive (adjusted RR: 1.32; 95% CI: 1.19, 1.47). The adjusted results were attenuated slightly but even after adjustment for potential confounders identified a priori, the results remained similar and statistically significant (Supplementary Table A1). To evaluate whether the association between ACEs and stress was explained fully or in part by obesity we ran a model adjusting for obesity. When adding obesity to the models for ACEs and all outcomes, associations were attenuated only slightly suggesting that obesity may not mediate the association between ACEs and stress (results not shown).

### The association between obesity and stress by ACEs

The tests for interaction by ACEs on both the additive and multiplicative scales for the association between obesity and stress are provided in Table 4. There was no consistent evidence of interaction on either the additive or multiplicative scale by ACEs on any of the outcomes. Only the multiplicative interaction between class III obesity and ACEs for stressors within the health domain was statistically significant (RERI = –0.34; 95% CI: –0.71, 0.02; RRR = 0.74; 95% CI: 0.58, 0.96), meaning among people with class III obesity, those with ACEs, compared to those with no ACEs, were less likely to report an additional stressor within the health domain.

**Table 4:** Adjusted relative risks^a^ (RRs) and 95% confidence Intervals (CIs) for the joint exposure of obesity and adverse childhood experiences (ACEs) among Canadian adults in the Canadian Longitudinal Study on Aging (CLSA) COVID-19 Questionnaire Exit Survey (September–December 2020) and interaction on the additive and multiplicative scales.

Health domain					
	Normal weight RR (95% CI)	Overweight RR (95% CI)	Obesity class I RR (95% CI)	Obesity class II RR (95% CI)	Obesity class III RR (95% CI)
No ACEs	1.00	1.04 (0.96, 1.14); *p* = 0.34	1.17 (1.05, 1.30); *p* = 0.004	1.06 (0.90, 1.23); *p* = 0.49	1.50 (1.24, 1.81); *p* < 0.0001
ACEs	1.31 (1.19, 1.44); *p* < 0.0001	1.38 (1.26, 1.50); *p* < 0.0001	1.49 (1.35, 1.64); *p* < 0.0001	1.54 (1.36, 1.76); *p* < 0.0001	1.47 (1.24, 1.73); *p* < 0.0001
Additive (RERI)^b^	REF	0.02 (-0.12, 0.17); *p* = 0.74	0.01 (–0.16, 0.19); *p* = 0.87	0.18 (-0.06, 0.42); *p* = 0.15	−0.34 (−0.71, 0.02); *p* = 0.07
Multiplicative (RRR)^c^	REF	1.01 (0.89, 1.14); *p* = 0.90	0.98 (0.85, 1.12); *p* = 0.73	1.12(0.92, 1.37); *p* = 0.27	0.74 (0.58, 0.96); *p* = 0.02
Resources domain					
	Normal weight RR (95% CI)	Overweight RR (95% CI)	Obesity class I RR (95% CI)	Obesity class II RR (95% CI)	Obesity class III RR (95% CI)
No ACEs	1.00	1.09 (1.00, 1.19); *p* = 0.06	1.21 (1.09, 1.24); *p* = 0.0003	1.35 (1.17, 1.55); *p* < 0.0001	1.38 (1.14, 1.66); *p* = 0.001
ACEs	1.35 (1.23, 1.48); *p* < 0.0001	1.37 (1.26, 1.50); *p* < 0.0001	1.58 (1.44, 1.74); *p* < 0.0001	1.68 (1.49, 1.89); *p* < 0.0001	1.78 (1.54, 2.06); *p* < 0.0001
Additive (RERI)	REF	–0.07 (–0.21, 0.08); *p* = 0.37	0.03 (-0.15, 0.20); *p* = 0.76	–0.02 (–0.28, 0.24); *p* = 0.88	0.05 (-0.30, 0.40); *p* = 0.78
Multiplicative (RRR)	REF	0.93 (0.83, 1.05); *p* = 0.26	0.97 (0.85, 1.11); *p* = 0.68	0.92 (0.77, 1.11); *p* = 0.38	0.96 (0.76, 1.21); *p* = 0.71
Relationships domain					
	Normal weight RR (95% CI)	Overweight RR (95% CI)	Obesity class I RR (95% CI)	Obesity class II RR (95% CI)	Obesity class III RR (95% CI)
No ACEs	1.00	0.98 (0.92, 1.05); *p* = 0.60	0.97 (0.89, 1.06); *p* = 0.50	1.07 (0.94, 1.20); *p* = 0.30	1.07 (0.90, 1.26); *p* = 0.43
ACEs	1.11 (1.03, 1.20); *p* = 0.01	1.17 (1.09, 1.25); *p* < 0.0001	1.19 (1.09, 1.25); p < 0.0001	1.12 (1.00, 1.25); *p* = 0.06	1.14 (0.98, 1.31); *p* = 0.08
Additive (RERI)	REF	0.08 (–0.2, 0.18); *p* = 0.13	0.11 (-0.2, 0.23); *p* = 0.09	–0.06 (–0.24, 0.12); *p* = 0.51	–0.04 (–0.29, 0.20); *p* = 0.72
Multiplicative (RRR)	REF	1.08 (0.97, 1.18); *p* = 0.15	1.10 (0.98, 1.24); *p* = 0.10	0.94 (0.80, 1.11); *p* = 0.49	0.96 (0.77, 1.19); *p* = 0.69
Caregiving domain					
	Normal weight RR (95% CI)	Overweight RR (95% CI)	Obesity class I RR (95% CI)	Obesity class II RR (95% CI)	Obesity class III RR (95% CI)
No ACEs	1.00	0.98 (0.92, 1.05); *p* = 0.60	0.97 (0.89, 1.06); *p* = 0.50	1.07 (0.94, 1.20); *p* = 0.30	1.07 (0.90, 1.25); *p* = 0.43
ACEs	1.11 (1.03, 1.20); *p* = 0.007	1.17 (1.09, 1.25); *p* < 0.0001	1.19 (1.09, 1.28); *p* < 0.0001	1.12 (1.00, 1.25); *p* = 0.06	1.14 (0.98, 1.31); *p* = 0.08
Additive (RERI)	REF	–0.08 (–0.29, 0.12); *p* = 0.42	0.04 (–0.20, 0.28); *p* = 0.76	–0.05 (–0.41, 0.30); *p* = 0.77	0.13 (–0.33, 0.59); *p* = 0.57
Multiplicative (RRR)	REF	0.92 (0.77, 1.10); *p* = 0.38	1.02 (0.83, 1.26); *p* = 0.84	0.93 (0.70, 1.24); *p* = 0.62	1.10 (0.74, 1.63); *p* = 0.65
Total number of stressors					
	Normal weight RR (95% CI)	Overweight RR (95% CI)	Obesity class I RR (95% CI)	Obesity class II RR (95% CI)	Obesity class III RR (95% CI)
No ACEs	1.00	1.03 (0.98, 1.08); *p* = 0.19	1.08 (1.02, 1.15); *p* = 0.01	1.14 (1.05, 1.24); *p* = 0.001	1.25 (1.11, 1.39); *p* = 0.0001
ACEs	1.23 (1.17, 1.30); *p* < 0.0001	1.28 (1.22, 1.34); *p* < 0.0001	1.38 (1.30, 1.45); *p* < 0.0001	1.39 (1.29, 1.50); *p* < 0.0001	1.42 (1.29, 1.55); *p* < 0.0001
Additive (RERI)	REF	0.01 (–0.07, 0.09); *p* = 0.78	0.06 (–0.03, 0.15); *p* = 0.21	0.01 (–0.12, 0.15); *p* = 0.84	–0.06 (–0.25, 0.12); *p* = 0.50
Multiplicative (RRR)	REF	1.00 (0.94, 1.07); *p* = 0.94	1.03 (0.95, 1.11); *p* = 0.47	0.99 (0.88, 1.10); *p* = 0.80	0.92 (0.80, 1.07); *p* = 0.27
Perceived consequences of the pandemic					
	Normal weight RR (95% CI)	Overweight RR (95% CI)	Obesity class I RR (95% CI)	Obesity class II RR (95% CI)	Obesity class III RR (95% CI)
No ACEs	1.00	0.96 (0.93, 1.01); *p* = 0.09	0.99 (0.94, 1.04); *p* = 0.63	0.98 (0.90, 1.05); *p* = 0.53	0.98 (0.88, 1.08); *p* = 0.74
ACEs	1.06 (1.02, 1.11); *p* = 0.006	1.03 (0.99, 1.08); *p* = 0.13	1.00 (0.95, 1.05); *p* = 0.91	1.05 (0.98, 1.12); *p* = 0.17	1.11 (1.02, 1.19); *p* = 0.01
Additive (RERI)	REF	0.003 (-0.05, 0.6); *p* = 0.91	–0.05 (–0.12, 0.02); *p* = 0.18	0.01 (−0.09, 0.11); *p* = 0.89	0.06 (−0.07, 0.20); *p* = 0.36
Multiplicative (RRR)	REF	1.01 (0.95, 1.06); *p* = 0.85	0.95 (0.89, 1.02); *p* = 0.19	1.01 (0.91, 1.11); *p* = 0.87	1.06 (0.93, 1.21); *p* = 0.38

*CI* confidence intervals, *RR* relative risk, *REF* reference.

^a^Adjusted for sex, age group, racial background, physical activity, household income, alcohol consumption and depression.

^b^Interaction on the additive scale using Relative Excess Risk due to Interaction (RERI); Standard error calculated using the delta method [41–43].

^c^Interaction on the multiplicative scale using Ratio of Relative Risks (RRR).

### Sex differences

The tests for interaction of obesity by sex on the additive and multiplicative scale are shown in Supplemental Table A3. There was consistent evidence of negative interaction on the multiplicative scale for class III obesity, such that females with class III obesity were less likely to report stress outcomes during the pandemic than males. For example, the joint exposure of having obesity class III and being female was associated with lower reports of stressors in the health domain (RERI = –0.27; 95% CI: –0.59, 0.05; RRR = 0.79; 95% CI: 0.63, 1.00), resources domain (RERI = –0.31; 95% CI: –0.61, –0.01; RRR = 0.80; 95% CI: 0.65, 0.98), caregiving domain (RERI = –0.60; 95% CI: –1.10, –0.09; RRR = 0.62; 95% CI: 0.44, 0.88), total stressors (RERI = –0.25; 95% CI: –0.43, –0.07; RRR = 0.80; 95% CI: 0.70, 0.91) and perceiving the pandemic as negative or very negative (RERI = 0.01; 95% CI: –0.07, 0.09; RRR = 0.89; 95% CI: 0.80, 0.99).The tests for interaction of ACEs by sex are shown in Supplemental Table A2. There was limited evidence of interaction on either scale, and all of the tests except one were not statistically significant. The only significant test for interaction was on the multiplicative scale for the perceived consequences of the pandemic such that, females with 4–8 ACEs were less likely to perceive the pandemic as negative or very negative compared to males (RRR = 0.91; 95% CI: 0.84, 0.99, *p* = 0.02).

### Sensitivity analysis

Sensitivity analyses to explore differences in associations between child maltreatment ACEs and measures of stress, and family dysfunction ACEs and measures of stress can be found in Table A4. For both maltreatment ACEs and family dysfunction ACEs, the greatest risk of reporting an additional stressor, or perceiving the consequences of the pandemic as negative/very negative were among those who reported the most ACEs. Associations were slightly larger for those who reported the most maltreatment ACEs.

## Discussion

The findings from this study contribute to our understanding of the experiences of stress among people with obesity during the COVID-19 pandemic and the cyclical relationship between obesity and stress. A dose-response association was found between obesity some measures of stress (total stressors, resources domain, and health domain). Although ACEs did not modify this association, it was found to be independently associated with stress experienced during the pandemic, as we identified a strong dose-response association between ACEs and all measures of stress. It was hypothesized that obesity may mediate the association between ACEs and stress experienced during the pandemic given the cyclical association stress and obesity share, however, the preliminary mediation analysis did not find obesity to be a mediator. This may be related to the timing of the measurement or that there are multiple pathways whereby ACEs influenced pandemic-related stress. Future research exploring the mechanisms behind these associations is needed, as it can be used to inform the response to future disasters or stressful events.

The joint exposures of obesity and sex were significant, meaning sex modifies these associations. Females with ACEs and with class III obesity were less likely to report an additional stressor compared to males. Our study appears to be the first to evaluate the joint effect of sex with other distal and proximal factors to stress caused by population-level adversity, such as the COVID-19 pandemic. However, studies that evaluated sex independently found females compared to males typically have higher reports of psychological related outcomes following disasters [19, 45].

The findings suggest that people with obesity were more likely to experience stressors during the pandemic, however, we found they were less likely to perceive the consequences of the pandemic as negative or very negative. Similarly, people who experienced increased adversity in childhood, had worse perceptions of experiences during the pandemic, which is consistent with the literature surrounding the psychological changes that occur after exposure to adversity extending beyond childhood and altering experiences later in life [8, 46]. Understanding the association both ACEs and obesity have with stress during the pandemic, can help to inform future screening programs that can identify who may be at the greatest risk of the worst outcomes or experiences during a stressful event. It is possible that we did not find ACEs to modify the association between obesity and stress during the pandemic due to the measures we used, the population within the study (e.g., community dwelling older adults), or the timing of assessment. For instance, we may have found effect modification by ACEs if we had additional measures of stress after the first year of the COVID-19 pandemic (after December 2020), as people’s response may have been different to the prolonged stress associated with the pandemic. Alternatively, it is possible that ACEs really does not modify this association and that stress experienced during the pandemic did not vary by experiences that occurred in early life among those with and without obesity. Future research is needed to understand why individuals with obesity experienced an increased risk of stress and worse perceptions of the pandemic. A potential pathway between obesity and these stressors could be related to weight bias and stigma; there was extensive media coverage highlighting obesity as a potential risk factor for COVID-19 mortality which may have increased weight stigma [16]. This information could be used to inform targeted strategies aimed at individuals who are overweight or have obesity, to help develop coping mechanisms, which in turn could break this cycle between obesity and stress. People with obesity may also have a greater stress response since obesity leads to a stimulation of the stress system within the body, including glucocorticoids or other stress hormones [1]. This activation may make them more susceptible to worse experiences. It is also possible that people who have had ACEs respond to stressful situations or events, such as the COVID-19 pandemic, differently. Following ACEs a person may differentially manage or respond to a stressful event making them more susceptible to a greater physiological or emotional stress response [47]. In addition, exposure to ACEs had been found to be linked to resilience, meaning the ability to overcome the negative experience, which has been found to lessen negative outcomes [48].

Strengths of this study include the availability of longitudinal data, which allowed for an assessment of both proximal (obesity in adulthood) and distal (ACEs) factors that are associated with stressors during the COVID-19 pandemic. This is one of the first studies to explore potential factors that may impact older adults’ experiences of stress during the pandemic, using a nationally generalizable cohort of over 23,000 participants. Another strength is the measurement of obesity, where most of the sample (65%) had obesity measured by a trained research assistant, and the remaining particpants (35%) self-reported obesity, which was corrected using validated correction factors to overcome biases associated with self-report. It is also a strength that we evaluated interaction on both the additive and multiplicative scales as recommended in the epidemiology methods literature [41]. The use of the additive scale provides important evidence for interaction from a causal perspective, as it explains if the presence of one exposure depends on the presence or absence of a second exposure [41, 42, 49]. The findings from this study are consistent with life course epidemiology frameworks that suggest an accumulation of risk can lead to increased disease later in life, as we identified measures of stress during the pandemic varied on both proximal and distal factors [25]. Limitations of this study include the sample demographics, as the current sample is primarily of white racial background which may limit the representativeness of findings. However, the demographics of the CLSA have been found to be similar to other nationally representative Canadian surveys and data from the Canadian census [26]. In addition, participants were asked to recall stressors, and perceptions of the consequences from the start of the pandemic at a relatively early period of the pandemic (September to December 2020). People’s experiences may have changed throughout the pandemic as the pandemic is still ongoing as of February 2022. Additional stressors may have also been experienced beyond the 12-items asked in the CLSA COVID-19 Questionnaire survey (e.g., loss of employment). Although the measures used in this study have previously been used in disaster research [18, 34–36] and were modified from gold-standard tools, it is a limitation that these tools have not been validated in the CLSA sample. Another potential limitation includes the use of self-reported recall for assessment of ACEs as this may introduce information bias, where individuals with the outcomes of interest recall past experiences differently. Given that CLSA COVID-19 Questionnaire participants were from both the Tracking and Comprehensive cohorts of the CLSA, data on BMI was collected differently (self-report versus measured). We addressed this issue by applying a correction factor to self-reported BMI to account for any biases associated with self-report [27]. It was a limitation that the correction factor used was from 2005, however this is currently the only equation available in the Canadian context to correct for self-reported BMI. We also did not have BMI measures at the time of the CLSA COVID-19 Questionnaire survey, so the use of BMI at CLSA follow-up 1 (2015–2018) may not necessarily reflect participants BMI at the time of the CLSA COVID-19 Exit Survey (Sept-Dec 2020). Finally, selection bias may also be a concern in this study given the age of the participants recruited in the CLSA, and the inability of some people in this age group to participate.

Overall, these findings may be important beyond the COVID-19 pandemic. We found people with obesity were more likely to report stressors but did not perceive the consequences of the pandemic as negative, whereas people who reported childhood adversity reported stressors and perceived the consequences of the pandemic as negative. These findings confirm different subgroups of people perceived themselves to be more susceptible to stress associated with a stressful event, such as the COVID-19 pandemic. These findings build on the framework proposed by van der Valk et al., [1] that outlines the relationship stress and obesity share, however, future research will be needed to further understand why people with obesity were more likely to report stressors but did not perceive the consequences of the pandemic as negative. The findings of this study are important beyond the COVID-19 pandemic, as it is apparent different subgroups are more susceptible to stress, which is likely to extend to other stressful events. Research will be needed to explore the long-term effects of stress experienced during the pandemic. Given the cyclical association that stress and obesity share, it is likely the pandemic will have lasting effects on future rates of obesity [50]. It will be important to determine how stress experienced during the pandemic impacts obesity rates, and potential mechanisms for this association, as it can be used to develop targeted interventions, including emotion regulation and coping strategies, helping to eliminate the cyclical association between stress and obesity, mitigating the burden of disease caused by obesity. The development of these interventions can be incorporated into clinical practice, where health professionals can identify those at the greatest risk, targeting health care to better meet their needs, improving overall health and wellbeing.

## Disclaimer

The opinions expressed in this manuscript are the author’s own and do not reflect the views of the Canadian Longitudinal Study on Aging or the Government of Canada.

## Supplementary information

Supplemental Information

## Data Availability

Data are available from the Canadian Longitudinal Study on Aging (www.clsa-elcv.ca) for researchers who meet the criteria for access to de-identified CLSA data.

## References

[1] van der Valk ES, Savas M, van Rossum EFC (2018). Stress and obesity: are there more susceptible individuals?. Curr Obes Rep.

[2] Salleh MOHDR (2008). Life event, stress and illness. Malays J Med Sci.

[3] Tomiyama AJ (2019). Stress and obesity. Annu Rev Psychol.

[4] Wiss DA, Brewerton TD (2020). Adverse childhood experiences and adult obesity: a systematic review of plausible mechanisms and meta-analysis of cross-sectional studies. Physiol Behav.

[5] Nurius PS, Green S, Logan-Greene P, Borja S (2015). Life course pathways of adverse childhood experiences toward adult psychological well-being: a stress process analysis. Child Abuse Neglect.

[6] Palmisano GL, Innamorati M, Vanderlinden J (2016). Life adverse experiences in relation with obesity and binge eating disorder: a systematic review. J Behav Addict.

[7] Duffy KA, McLaughlin KA, Green PA (2018). Early life adversity and health-risk behaviors: proposed psychological and neural mechanisms. Ann NY Acad Sci.

[8] Riem MME, Karreman A (2019). Childhood adversity and adult health: the role of developmental timing and associations with accelerated aging. Child Maltreat.

[9] Franke HA (2014). Toxic stress: effects, prevention and treatment. Children..

[10] Hale T, Angrist N, Goldszmidt R, Kira B, Petherick A, Phillips T (2021). A global panel database of pandemic policies (Oxford COVID-19 Government Response Tracker). Nat Hum Behav.

[11] Leigh JP, Fiest K, Brundin-Mather R, Plotnikoff K, Soo A, Sypes EE (2020). A national cross-sectional survey of public perceptions of the COVID-19 pandemic: Self-reported beliefs, knowledge, and behaviors. PLOS ONE.

[12] Park CL, Russell BS, Fendrich M, Finkelstein-Fox L, Hutchison M, Becker J (2020). Americans’ COVID-19 stress, coping, and adherence to CDC guidelines. J Gen Intern Med.

[13] De Rubeis V, Anderson LN, Khattar J, de Groh M, Jiang Y, Oz UE, et al. Stressors and perceived consequences of the COVID-19 pandemic among older adults in the Canadian Longitudinal Study on Aging (CLSA). CMAJ Open. 2022;10:E721–30. 10.9778/cmajo.20210313 PMC9377549 35944921

[14] Kompaniyets L Body Mass Index and Risk for COVID-19–Related Hospitalization, Intensive Care Unit Admission, Invasive Mechanical Ventilation, and Death — United States, March–December 2020. MMWR Morb Mortal Wkly Rep [Internet]. 2021;70. Available from: https://www.cdc.gov/mmwr/volumes/70/wr/mm7010e4.htm. 10.15585/mmwr.mm7010e4 PMC7951819 33705371

[15] Pearl RL, Schulte EM. Weight bias during the COVID-19 pandemic. Curr Obes Rep. 2021;10:1–10. 10.1007/s13679-021-00432-2 PMC7971403 33738699

[16] Puhl RM, Lessard LM, Larson N, Eisenberg ME, Neumark-Stzainer D (2020). Weight stigma as a predictor of distress and maladaptive eating behaviors during COVID-19: longitudinal findings from the EAT study. Ann Behav Med.

[17] De Rubeis V, Lee J, Anwer MS, Yoshida-Montezuma Y, Andreacchi AT, Stone E (2021). Impact of disasters, including pandemics, on cardiometabolic outcomes across the life-course: a systematic review. BMJ Open.

[18] King S, Laplante DP (2005). The effects of prenatal maternal stress on children’s cognitive development: project ice storm. Stress..

[19] Wahlström L, Michélsen H, Schulman A, Backheden M (2010). Childhood life events and psychological symptoms in adult survivors of the 2004 tsunami. Nordic Journal of Psychiatry.

[20] Guo J, Fu M, Liu D, Zhang B, Wang X, van IJzendoorn MH (2020). Is the psychological impact of exposure to COVID-19 stronger in adolescents with pre-pandemic maltreatment experiences? A survey of rural Chinese adolescents. Child Abuse Negl.

[21] Doom JR, Seok D, Narayan AJ, Fox KR. Adverse and benevolent childhood experiences predict mental health during the COVID-19 pandemic. Advers Resil Sci. 2021;2:1–12. 10.1007/s42844-021-00038-6 PMC8062213 33907733

[22] Gewirtz-Meydan A, Lassri D. A profile analysis of COVID-19 stress-related reactions: The importance of early childhood abuse, psychopathology, and interpersonal relationships. Child Abuse Negl. 2021;130:105442. 10.1016/j.chiabu.2021.105442 PMC8666322 34920898

[23] Statistics Canada. Overweight and obese adults, 2018;2019:8.

[24] Haahr-Pedersen I, Perera C, Hyland P, Vallières F, Murphy D, Hansen M (2020). Females have more complex patterns of childhood adversity: implications for mental, social, and emotional outcomes in adulthood. Eur J Psychotraumatol.

[25] Kuh D (2003). Life course epidemiology. J Epidemiol Community Health.

[26] Raina P, Wolfson C, Kirkland S, Griffith LE, Balion C, Cossette B (2019). Cohort Profile: The Canadian Longitudinal Study on Aging (CLSA). Int J Epidemiol.

[27] Shields M, Connor Gorber S, Janssen I, Tremblay MS (2011). Bias in self-reported estimates of obesity in Canadian health surveys: an update on correction equations for adults. Health Rep.

[28] Connor Gorber S, Shields M, Tremblay MS, McDowell I (2008). The feasibility of establishing correction factors to adjust self-reported estimates of obesity. Health Rep.

[29] Weir CB, Jan A BMI Classification percentile and cut off points. In: StatPearls [Internet]. Treasure Island (FL): StatPearls Publishing; 2020. Available from: http://www.ncbi.nlm.nih.gov/books/NBK541070/. 31082114

[30] Aronne LJ (2002). Classification of obesity and assessment of obesity-related health risks. Obesity Res.

[31] Joshi D, Raina P, Tonmyr L, MacMillan HL, Gonzalez A (2021). Prevalence of adverse childhood experiences among individuals aged 45 to 85 years: a cross-sectional analysis of the Canadian Longitudinal Study on Aging. CMAJ Open.

[32] Mian O, Anderson LN, Belsky DW, Gonzalez A, Ma J, Sloboda DM, et al. Associations of adverse childhood experiences with frailty in older adults: a cross-sectional analysis of data from the Canadian Longitudinal Study on Aging. Gerontology. 2021;68:1–10. 10.1159/000520327 34875667

[33] Bethell CD, Carle A, Hudziak J, Gombojav N, Powers K, Wade R (2017). Methods to assess adverse childhood experiences of children and families: toward approaches to promote child well-being in policy and practice. Acad Pediatr.

[34] Dancause KN, Veru F, Andersen RE, Laplante DP, King S. Prenatal stress due to a natural disaster predicts insulin secretion in adolescence. Early Hum Dev [Internet]. 2013;89:773–6. https://www.ncbi.nlm.nih.gov/pmc/articles/PMC3855052/. 10.1016/j.earlhumdev.2013.06.006 PMC3855052 23830724

[35] Cao-Lei L, Elgbeili G, Massart R, Laplante DP, Szyf M, King S (2015). Pregnant women’s cognitive appraisal of a natural disaster affects DNA methylation in their children 13 years later: project Ice Storm. Translational Psychiatry.

[36] Laplante DP, Brunet A, Schmitz N, Ciampi A, King S (2008). Project ice storm: prenatal maternal stress affects cognitive and linguistic functioning in 5½-year-old children. J Am Acad Child Adolesc Psychiatry.

[37] Abdalla SM, Ettman CK, Cohen GH, Galea S (2021). Mental health consequences of COVID-19: a nationally representative cross-sectional study of pandemic-related stressors and anxiety disorders in the USA. BMJ Open.

[38] Washburn RA, Smith KW, Jette AM, Janney CA (1993). The physical activity scale for the elderly (PASE): Development and evaluation. J Clin Epidemiol.

[39] Global Recommendations on Physical Activity for Health [Internet]. Geneva: World Health Organization; 2010. (WHO Guidelines Approved by the Guidelines Review Committee). Available from: http://www.ncbi.nlm.nih.gov/books/NBK305057/.

[40] Björgvinsson T, Kertz SJ, Bigda-Peyton JS, McCoy KL, Aderka IM (2013). Psychometric properties of the CES-D-10 in a psychiatric sample. Assessment..

[41] Knol MJ, VanderWeele TJ (2012). Recommendations for presenting analyses of effect modification and interaction. Int J Epidemiol.

[42] Shiba K, Kubzansky LD, Williams DR, VanderWeele TJ, Kim ES (2021). Associations between purpose in life and mortality by SES. Am J Prev Med.

[43] VanderWeele TJ, Knol MJ. A Tutorial on interaction. Epidemiologic Methods [Internet]. 2014;3:33–72. Available from: https://www.degruyter.com/document/doi/10.1515/em-2013-0005/html.

[44] Raina P, Wolfson C, Griffith LE, Kirkland S, MacMillian J, Basta N, et al. The impact of the COVID-19 pandemic on the mental health of older adults: a longitudinal analysis from the Canadian Longitudinal Study on Aging. Nature. 2021;1:1137–47. 10.1038/s43587-021-00128-1 37117519

[45] Bonanno GA, Galea S, Bucciarelli A, Vlahov D (2007). What predicts psychological resilience after disaster? The role of demographics, resources, and life stress. J Consult Clin Psychol.

[46] Herzog JI, Schmahl C (2018). Adverse childhood experiences and the consequences on neurobiological, psychosocial, and somatic conditions across the lifespan. Front Psychiatry.

[47] Sheffler JL, Piazza JR, Quinn JM, Sachs-Ericsson NJ, Stanley IH (2019). Adversechildhood experiences and coping strategies: identifying pathways to resiliencyin adulthood. Anxiety Stress Coping.

[48] Soleimanpour S, Geierstanger S, Brindis CD (2017). Adverse childhood experiences and resilience: addressing the unique needs of adolescents. Acad Pediatr.

[49] Corraini P, Olsen M, Pedersen L, Dekkers OM, Vandenbroucke JP (2017). Effect modification, interaction and mediation: an overview of theoretical insights for clinical investigators. Clin Epidemiol.

[50] Bakaloudi DR, Barazzoni R, Bischoff SC, Breda J, Wickramasinghe K, Chourdakis M (2022). Impact of the first COVID-19 lockdown on body weight: a combined systematic review and a meta-analysis. Clin Nutr.

