# Supplementary material for: Obesity and adverse childhood experiences in relation to stress during the COVID-19 pandemic: an analysis of the Canadian Longitudinal Study on Aging
Source: Int J Obes (Lond). 2023 Jan 23;47(3):197–206. doi: 10.1038/s41366-023-01258-9 (PMC9868513; doi:10.1038/s41366-023-01258-9)
Supplement: Supplementary file 1 — Supplemental Information [file 41366_2023_1258_MOESM1_ESM.docx]

**Supplemental Information**

**Table A1.** Unadjusted relative risks (RRs) and 95% confidence intervals (CIs) for the associations between adverse childhood experiences (ACEs), obesity and measures of stress during the COVID-19 pandemic among older adults in the Canadian Longitudinal Study on Aging (CLSA) COVID-19 Questionnaire Exit Survey

|  | **Stressor domains** | | | | **Total number**  **of stressors** | **Perceived consequences of the pandemic** |
| --- | --- | --- | --- | --- | --- | --- |
|  | **Health** | **Resources** | **Relationships** | **Caregiving** |  |  |
|  | Unadjusted RR (95% CI) | Unadjusted RR (95% CI) | Unadjusted RR (95% CI) | Unadjusted RR (95% CI) | Unadjusted RR (95% CI) | Unadjusted RR (95% CI) |
| **Number of ACEs** | 1.00  1.18 (1.12, 1.24)  1.25 (1.17, 1.32)  1.42 (1.33, 1.52)  1.51 (1.41, 1.61) |  |  |  |  |  |
| 0 |  | 1.00 | 1.00 | 1.00 | 1.00 | 1.00 |
| 1 |  | 1.11 (1.05, 1.16) | 1.09 (1.04, 1.13) | 1.12 (1.04, 1.20) | 1.11 (1.09, 1.15) | 1.10 (1.03, 1.17) |
| 2 |  | 1.24 (1.17, 1.31) | 1.15 (1.09, 1.21) | 1.18 (108, 1.29) | 1.20 (1.16, 1.24) | 1.09 (1.01, 1.19) |
| 3 |  | 1.42 (1.33, 1.52) | 1.21 (1.14, 1.28) | 1.31 (1.1, 1.45) | 1.33 (1.27, 1.38) | 1.26 (1.14, 1.39) |
| 4-8 |  | 1.74 (1.64, 1.85) | 1.36 (1.29, 1.44) | 1.63 (1.48, 1.80) | 1.53 (1.47, 1.59) | 1.37 (1.24, 1.51) |
| **Obesity** | 1.00  0.99 (0.94, 1.04)  1.11 (1.05, 1.17)  1.15 (1.06, 1.25)  1.32 (1.19, 1.46) |  |  |  |  |  |
| Normal weight |  | 1.00 | 1.00 | 1.00 | 1.00 | 1.00 |
| Overweight |  | 1.02 (0.98, 1.08) | 0.96 (0.92, 1.00) | 0.93 (0.87, 1.00) | 0.97 (0.95, 1.01) | 0.98 (0.95, 1.00) |
| Obesity Class I |  | 1.22 (1.15, 1.29) | 1.00 (0.95, 1.05) | 0.96 (0.88, 1.05) | 1.08 (1.04, 1.11) | 0.97 0.94, 1.00) |
| Obesity Class II |  | 1.37 (1.27, 1.48) | 1.03 (0.96, 1.10) | 1.07 (0.95, 1.21) | 1.15 (1.10, 1.21) | 1.00 (0.96, 1.04) |
| Obesity Class III |  | 1.58 (1.43, 1.73) | 1.09 (1.00, 1.19) | 1.12 (0.95, 1.21) | 1.27 (1.20, 1.35) | 1.07 (1.01, 1.12) |

CI: Confidence Intervals; RR: Relative risk

**Table A2.** Adjusted relative risks^1^ (RRs) and 95% confidence Intervals (CIs) for the joint exposure of adverse childhood experiences (ACEs) and sex among Canadian adults in the Canadian Longitudinal Study on Aging (CLSA) COVID-19 Questionnaire Exit Survey (September-December 2020) and effect modification on the additive and multiplicative scales

| **Health Domain** | | | | | |
| --- | --- | --- | --- | --- | --- |
|  | 0  RR (95% CI) | 1  RR (95% CI) | 2  RR (95% CI) | 3  RR (95% CI) | 4-8  RR (95% CI) |
| Male | 1.00 | 1.24 (1.15, 1.34); p<0.0001 | 1.23 (1.12, 1.35); p<0.0001 | 1.43 (1.28, 1.59); p<0.0001 | 1.46 (1.30, 1.59); p<0.0001 |
| Female | 1.20 (1.12, 1.29); p<0.0001 | 1.34 (1.23, 1.44); p<0.0001 | 1.46 (1.24, 1.59); p<0.0001 | 1.60 (1.45, 1.77); p<0.0001 | 1.62 (1.48, 1.78); p<0.0001 |
| Additive (RERI) ^2^ | REF | -0.10 (-0.23, 0.03); p=0.12 | 0.03 (-0.13, 0.18); p=0.73 | -0.03 (-0.23, 0.17); p=0.77 | -0.04 (-0.25, 0.16); p=0.69 |
| Multiplicative (RRR) ^3^ | REF | 0.90 (0.81, 1.00); p=0.05 | 0.99 (0.87, 1.12); p=0.84 | 0.93 (0.81, 1.07); p=0.33 | 0.92 (0.80, 1.07); p=0.28 |
| **Resources Domain** | | | | | |
|  | 0  RR (95% CI) | 1  RR (95% CI) | 2  RR (95% CI) | 3  RR (95% CI) | 4-8  RR (95% CI) |
| Male | 1.00 | 1.07 (1.00, 1.16); p=0.06 | 1.17 (1.07, 1.27); p-0.0004 | 1.34 (1.21, 1.48); p<0.0001 | 1.63 (1.47, 1.80); p<0.0001 |
| Female | 0.99 (0.93, 1.06); | 1.07 (1.00, 1.06); p=0.86 | 1.16 (1.06, 1.26); p=0.001 | 1.26 (1.15, 1.39); p<0.0001 | 1.46 (1.34, 1.58); p<0.0001 |
| Additive (RERI) | REF | 0.004 (-0.10, 0.11); p=0.94 | -0.01 (-0.14, 0.13); p=0.93 | -0.08 (-0.25, 0.09); p=0.38 | -0.17 (-0.35, 0.02); p=0.08 |
| Multiplicative (RRR) | REF | 1.00 (0.91, 1.11); p=0.94 | 1.00 (0.88, 1.12); p=0.95 | 0.94 (0.82, 1.08); p=0.41 | 0.90 (0.79, 1.02); p=0.11 |
| **Relationships Domain** | | | | | |
|  | 0  RR (95% CI) | 1  RR (95% CI) | 2  RR (95% CI) | 3  RR (95% CI) | 4-8  RR (95% CI) |
| Male | 1.00 | 1.09 (1.02, 1.16); p=0.01 | 1.11 (1.03, 1.20); p=0.008 | 1.20 (1.09, 1.21); p=0.0002 | 1.29 (1.16, 1.42); p<0.0001 |
| Female | 1.30 (1.23, 1.38); p<0.0001 | 1.37 (1.29, 1.46); p<0.0001 | 1.43 (1.34, 1.54); p<0.0001 | 1.45 (1.34, 1.58); p<0.0001 | 1.59 (1.47, 1.71); p<0.0001 |
| Additive (RERI) | REF | -0.02 (-0.12, 0.09); p=0.73 | 0.02 (-0.11, 0.14); p=0.77 | -0.04 (-0.20, 0.11); p=0.60 | 0.002 (-0.16, 0.17); p=0.98 |
| Multiplicative (RRR) | REF | 0.97 (0.89, 1.06); p=0.47 | 0.99 (0.89, 1.10); p=0.84 | 0.93 (0.83, 1.06); p=0.28 | 0.95 (0.84, 1.07); p=0.41 |
| **Caregiving Domain** | | | | | |
|  | 0  RR (95% CI) | 1  RR (95% CI) | 2  RR (95% CI) | 3  RR (95% CI) | 4-8  RR (95% CI) |
| Male | 1.00 | 1.13 (1.00, 1.27); p=0.05 | 1.10 (0.95, 1.28); p=0.19 | 1.28 (1.08, 1.52); p=0.004 | 1.55 (1.30, 1.83); p<0.0001 |
| Female | 1.38 (1.24, 1.54); p<0.0001 | 1.47 (1.32, 1.65); p<0.0001 | 1.58 (1.39, 1.79); p<0.0001 | 1.63 (1.40, 1.88); p<0.0001 | 1.92 (1.68, 2.19) p<0.0001 |
| Additive (RERI) | REF | -0.03 (-0.26, 0.18); p=0.75 | 0.09 (-0.15, 0.33); p=0.45 | -0.04 (-0.34, 0.27); p=0.82 | -0.01 (-0.33, 0.32); p=0.96 |
| Multiplicative (RRR) | REF | 0.95 (0.81, 1.11); p=0.49 | 1.03 (0.86, 1.25); p=0.73 | 0.92 (0.74, 1.15); p=0.45 | 0.90 (0.73, 1.11); p=0.31 |
| **Total number of stressors** | | | | | |
|  | 0  RR (95% CI) | 1  RR (95% CI) | 2  RR (95% CI) | 3  RR (95% CI) | 4-8  RR (95% CI) |
| Male | 1.00 | 1.12 (1.08, 1.17); p<0.0001 | 1.16 (1.10, 1.22); p<0.0001 | 1.31 (1.23, 1.39); p<0.0001 | 1.46 (1.37, 1.56); p<0.0001 |
| Female | 1.20 (1.15, 1.24); p<0.0001 | 1.29 (1.23, 1.34); p<0.0001 | 1.37 (1.31, 1.44); p<0.0001 | 1.45 (1.38, 1.54); p<0.0001 | 1.60 (1.52, 1.68) p<0.0001 |
| Additive (RERI) | REF | 0.01 (-0.04, 0.06); p=0.64 | 0.02 (-0.04, 0.08); p=0.48 | -0.02 (-0.09, 0.06); p=0.68 | -0.03 (-0.10, 0.05); p=0.47 |
| Multiplicative (RRR) | REF | 1.01 (0.96, 1.07); p=0.63 | 1.02 (0.96, 1.09); p=0.48 | 0.99 (0.92, 1.06); p=0.70 | 0.98 (0.91, 1.05); p=0.49 |
| **Perceived consequences of the pandemic** | | | | | |
|  | 0  RR (95% CI) | 1  RR (95% CI) | 2  RR (95% CI) | 3  RR (95% CI) | 4-8  RR (95% CI) |
| Male | 1.00 | 1.02 (0.98, 1.06); p=0.33 | 1.02 (0.97, 1.06); p=0.41 | 1.08 (1.02, 1.12); p=0.003 | 1.12 (1.06, 1.18); p<0.0001 |
| Female | 0.99 (0.96, 1.02); p=0.57 | 1.02 (0.98, 1.06); p=0.27 | 1.03 (0.98, 1.08); p=0.16 | 1.05 (1.00, 1.11); p=0.04 | 1.08 (1.03, 1.13); p=0.001 |
| Additive (RERI) | REF | -0.03 (-0.10, 0.04); p=0.34 | 0.02 (-0.06, 0.11); p=0.62 | -0.05 (-0.16, 0.06); p=0.38 | -0.06 (-0.18, 0.05); p=0.28 |
| Multiplicative (RRR) | REF | 0.96 (0.90, 1.02); p=0.14 | 0.99 (0.93, 1.07); p=0.85 | 0.93 (0.86, 1.01); p=0.08 | 0.91 (0.84, 0.99); p=0.02 |

CI: Confidence Intervals; RR: Relative risk; REF: Reference

1. Adjusted for sex, age group, racial background, physical activity, household income, alcohol consumption and depression
2. Effect modification on the additive scale using Relative Excess Risk due to Interaction (RERI); Standard error calculated using the delta method ^36–38^
3. Effect modification on the multiplicative scale using Ratio of Relative Risks (RRR)

**Table A3.** Adjusted relative risks^1^ (RRs) and 95% confidence Intervals (CIs) for the joint exposure of obesity and sex among Canadian adults in the Canadian Longitudinal Study on Aging (CLSA) COVID-19 Questionnaire Exit Survey (September-December 2020) and effect modification on the additive and multiplicative scales

| **Health Domain** | | | | | |
| --- | --- | --- | --- | --- | --- |
|  | Normal weight  RR (95% CI) | Overweight  RR (95% CI) | Obesity class I  RR (95% CI) | Obesity class II  RR (95% CI) | Obesity class III  RR (95% CI) |
| Male | 1.00 | 1.06 (0.98, 1.15); p=0.17 | 1.19 (1.08, 1.31); p=0.0003 | 1.09 (0.95, 1.26); p=0.21 | 1.47 (1.22, 1.76); p<0.0001 |
| Female | 1.21 (1.12, 1.32); p<0.0001 | 1.23 (1.13, 1.34); p<0.0001 | 1.33 (1.21, 1.46); p<0.0001 | 1.41 (1.26, 1.59); p<0.0001 | 1.42 (1.23, 1.63); p<0.0001 |
| Additive (RERI) ^2^ | REF | -0.05 (-0.17, 0.07); p=0.45 | -0.07 (-0.22, 0.07); p=0.32 | 0.11 (-0.10, 0.31); p=0.31 | -0.27 (-0.59, 0.05); p=0.10 |
| Multiplicative (RRR) ^3^ | REF | 0.95 (0.86, 1.06); p=0.39 | 0.92 (0.81, 1.04); p=0.18 | 1.06 (0.89, 1.27); p=0.48 | 0.79 (0.63, 1.00); p=0.04 |
| **Resources Domain** | | | | | |
|  | Normal weight  RR (95% CI) | Overweight  RR (95% CI) | Obesity class I  RR (95% CI) | Obesity class II  RR (95% CI) | Obesity class III  RR (95% CI) |
| Male | 1.00 | 1.08 (1.00, 1.17); p=0.05 | 1.24 (1.14,1.36); p<0.0001 | 1.37 (1.21, 1.54); p<0.0001 | 1.60 (1.36, 1.88); p<0.0001 |
| Female | 1.04 (0.96, 1.13); p=0.03 | 1.08 (1.00, 1.17); p=0.05 | 1.23 (1.13, 1.35); p<0.0001 | 1.34 (1.20, 1.50); p<0.0001 | 1.34 (1.17, 1.52); p<0.0001 |
| Additive (RERI) | REF | -0.04 (-0.15, 0.07); p=0.45 | -0.05 (-0.19, 0.08); p=0.44 | -0.07 (-0.27, 0.13); p=0.48 | -0.31 (-0.61, -0.01); p=0.04 |
| Multiplicative (RRR) | REF | 0.96 (0.87, 1.06); p=0.44 | 0.95 (0.85, 1.07); p=0.40 | 0.94 (0.80, 1.10); p=0.42 | 0.80 (0.65, 0.98); p=0.03 |
| **Relationships Domain** | | | | | |
|  | Normal weight  RR (95% CI) | Overweight  RR (95% CI) | Obesity class I  RR (95% CI) | Obesity class II  RR (95% CI) | Obesity class III  RR (95% CI) |
| Male | 1.00 | 0.99 (0.93, 1.06); p=0.86 | 1.04 (.96, 1.12); p=0.34 | 0.99 (0.97, 1.11); p=0.82 | 1.13 (0.95, 1.34); p=0.16 |
| Female | 1.27 (1.19, 1.36); p<0.0001 | 1.30 (1.21, 1.39); p<0.0001 | 1.32 (1.22, 1.43); p<0.0001 | 1.35 (1.23, 1.49); p<0.0001 | 1.29 (1.14, 1.49); p<0.0001 |
| Additive (RERI) | REF | 0.03 (-0.07, 0.13); p=0.55 | 0.01 (-0.11, 0.13); p=0.87 | 0.09 (-0.07, 0.26); p=0.27 | -0.12 (-0.36, 0.12); p=0.33 |
| Multiplicative (RRR) | REF | 1.02 (0.94, 1.12); p=0.58 | 1.00 (0.90, 1.11); p=0.98 | 1.08 (0.93, 1.25); p=0.33 | 0.89 (0.73, 1.10); p=0.27 |
| **Caregiving Domain** | | | | | |
|  | Normal weight  RR (95% CI) | Overweight  RR (95% CI) | Obesity class I  RR (95% CI) | Obesity class II  RR (95% CI) | Obesity class III  RR (95% CI) |
| Male | 1.00 | 1.06 (0.93, 1.21); p=0.38 | 1.15 (0.99, 1.33); p=0.06 | 1.13 (0.91, 1.40); p=0.26 | 1.50 (1.13, 1.97); p=0.004 |
| Female | 1.48 (1.30, 1.69); p<0.0001 | 1.47 (1.30, 1.67); p<0.0001 | 1.44 (1.25, 1.66); p<0.0001 | 1.65 (1.38, 1.96); p<0.0001 | 1.38 (1.10, 1.71), p=0.004 |
| Additive (RERI) | REF | -0.06 (-0.26, 0.13); p=0.52 | -0.19 (-0.43, 0.05); p=0.13 | 0.04 (-0.29, 0.38); p=0.80 | -0.60 (-1.10, -0.09); p=0.02 |
| Multiplicative (RRR) | REF | 0.94 (0.80, 1.10); p=0.46 | 0.85 (0.70, 1.02); p=0.08 | 0.99 (0.76, 1.29); p=0.93 | 0.62 (0.44, 0.88) p=0.01 |
| **Total number of stressors** | | | | | |
|  | Normal weight  RR (95% CI) | Overweight  RR (95% CI) | Obesity class I  RR (95% CI) | Obesity class II  RR (95% CI) | Obesity class III  RR (95% CI) |
| Male | 1.00 | 1.04 (0.99, 1.09); p=0.09 | 1.15 (1.09, 1.21); p<0.0001 | 1.14 (1.05, 1.23); p=0.001 | 1.39 (1.24, 1.54); p<0.0001 |
| Female | 1.21 (1.16, 1.27); p<0.0001 | 1.24 (1.18, 1.30); p<0.0001 | 1.31 (1.24, 1.28); p<0.0001 | 1.39 (1.30, 1.49); p<0.0001 | 1.35 (1.24, 1.46); p<0.0001 |
| Additive (RERI) | REF | -0.02 (-0.08, 0.05); p=0.59 | -0.05 (-0.13, 0.03); p=0.25 | 0.04 (-0.07, 0.16); p=0.48 | -0.25 (-0.43, -0.07); p=0.09 |
| Multiplicative (RRR) | REF | 0.98 (0.92, 1.04); p=0.48 | 0.94 (0.88, 1.02); p=0.10 | 1.01 (0.92, 1.11); p=0.86 | 0.80 (0.70, 0.91); p=0.0009 |
| **Perceived consequences of the pandemic** | | | | | |
|  | Normal weight  RR (95% CI) | Overweight  RR (95% CI) | Obesity class I  RR (95% CI) | Obesity class II  RR (95% CI) | Obesity class III  RR (95% CI) |
| Male | 1.00 | 0.96 (0.93, 1.00); p=0.05 | 0.96 (0.92, 1.00); p=0.10 | 0.99 (0.93, 1.06); p=0.86 | 1.14 (1.04, 1.22); p=0.002 |
| Female | 0.99 (0.95, 1.03); p=0.48 | 0.96 (0.93, 1.00); p=0.07 | 0.96 (.92, 1.00); p=0.09 | 0.99 (0.93, 1.05); p=0.74 | 1.00 (0.93, 1.07); p=0.92 |
| Additive (RERI) | REF | 0.02 (-0.03, 0.06); p=0.54 | 0.01 (-0.05, 0.07); p=0.74 | 0.01 (-0.07, 0.09); p=0.82 | 0.01 (-0.07, 0.09); p=0.82 |
| Multiplicative (RRR) | REF | 1.02 (0.97, 1.07); p=0.55 | 1.01 (0.95, 1.07); p=0.75 | 1.01 (0.93, 1.10); p=0.83 | 0.89 (0.80, 0.99); p=0.03 |

CI: Confidence Intervals; RR: Relative risk; REF: Reference

1. Adjusted for sex, age group, racial background, physical activity, household income, alcohol consumption and depression
2. Effect modification on the additive scale using Relative Excess Risk due to Interaction (RERI); Standard error calculated using the delta method ^36–38^
3. Effect modification on the multiplicative scale using Ratio of Relative Risks (RRR)

**Table A4.** Sensitivity analysis exploring the association between maltreatment ACEs and measures of stress during the COVID-19 pandemic, and family dysfunction ACEs and measures of stress during the COVID-19 pandemic among older adults in the Canadian Longitudinal Study on Aging (CLSA) COVID-19 Questionnaire Exit Survey

|  | **Stressor domains** | | | | **Total number**  **of stressors** | **Perceived consequences of the pandemic** |
| --- | --- | --- | --- | --- | --- | --- |
|  | **Health** | **Resources** | **Relationships** | **Caregiving** |  |  |
|  | Adjusted RR (95% CI)^1^ | Adjusted RR (95% CI)^1^ | Adjusted RR (95% CI)^1^ | Adjusted RR (95% CI)^1^ | Adjusted RR (95% CI)^1^ | Adjusted RR (95% CI)^1^ |
| **Number of maltreatment ACEs** | 1.00  1.18 (1.11-1.24)  1.20 (1.12-1.28)  1.30 (1.20-1.40)  1.53 (1.36-1.71) |  |  |  |  |  |
| 0 |  | 1.00 | 1.00 | 1.00 | 1.00 | 1.00 |
| 1 |  | 1.12 (1.06-1.17) | 1.10 (1.06-1.15) | 1.12 (1.04-1.21) | 1.13 (1.10-1.16) | 1.13 (1.06-1.22) |
| 2 |  | 1.27 (1.19-1.35) | 1.14 (1.08-1.20) | 1.22 (1.11-1.33) | 1.20 (1.15-1.24) | 1.20 (1.10-1.32) |
| 3 |  | 1.41 (1.31-1.52) | 1.21 (1.14-1.29) | 1.22 (1.09-1.36) | 1.29 (1.23-1.34) | 1.39 (1.25-1.56) |
| 4 |  | 1.67 (1.49-1.84) | 1.27 (1.15-1.40) | 1.57 (1.34-1.83) | 1.47 (1.38-1.57) | 1.22 (1.02-1.46) |
| **Number of family dysfunction ACEs** | 1.00  1.12 (1.07-1.18)  1.23 (1.14-1.33)  1.36 (1.11-1.65) |  |  |  |  |  |
| 0 |  | 1.00 | 1.00 | 1.00 | 1.00 | 1.00 |
| 1 |  | 1.09 (1.04-1.14) | 1.05 (1.01-1.09) | 1.13 (1.06-1.21) | 1.09 (1.06-1.11) | 1.03 (0.97-1.10) |
| 2 |  | 1.23 (1.14-1.32) | 1.15 (1.08-1.22) | 1.15 (1.03-1.28) | 1.19 (1.14-1.24) | 1.06 (0.95-1.19) |
| 3 |  | 1.39 (1.15-1.66) | 1.15 (0.97-1.35) | 1.63 (1.26-2.07) | 1.33 (1.19-1.48) | 1.17 (0.86-1.61) |

CI: Confidence Intervals; RR: Relative risk

1. Adjusted for sex, age group, racial background, physical activity, household income, alcohol consumption and depression
2. Maltreatment ACEs include physical abuse, sexual abuse, emotional abuse, neglect, intimate partner violence
3. Family dysfunction ACEs include parental divorce/separation, living with a family member with mental health problems, death of a parent
